# The association between a dietary index for the gut microbiota and frailty in older adults: emphasising the mediating role of inflammatory indicators

**DOI:** 10.3389/fnut.2025.1562278

**Published:** 2025-07-09

**Authors:** Huangyi Yin, Yue Qiu, Chaomei Gan, Yubo Zhou, Tingting Chen, Min Liang

**Affiliations:** ^1^Geriatric Endocrinology, The First Affiliated Hospital of Guangxi Medical University, Nanning, China; ^2^Osteoarticular Surgery, The First Affiliated Hospital of Guangxi Medical University, Nanning, China

**Keywords:** gut microbiome, frailty, DI-GM, NHANES, ageing

## Abstract

**Background:**

The dietary index for the gut microbiota (DI-GM) is a novel indicator of gut microbiome health, and its association with frailty remains unknown.

**Methods:**

We recruited participants from the 2007–2020 National Health and Nutrition Examination Survey (NHANES). Restricted cubic splines (RCSs) and multifactorial logistic regression were employed to investigate the relationship between the DI-GM and frailty. A mediation analysis was conducted to evaluate the mediating role of inflammatory markers. Stratification and sensitivity analyses were performed to evaluate the consistency of this association.

**Results:**

A total of 4,578 eligible individuals were screened, with a frailty prevalence of 35.50%. After adjusting for all of the covariates, each unit increase in the DI-GM was associated with a 6% decrease in the prevalence of frailty (OR = 0.94; 95% CI: 0.89, 0.99; *p* = 0.020). Furthermore, participants in the highest tertile of the DI-GM were significantly less likely to be frail than were those in the lowest tertile (OR = 0.70; 95% CI: 0.53, 0.91; *p* = 0.008). Mediation analysis revealed that inflammatory biomarkers significantly mediated the association between the DI-GM and frailty, with percentages of 16.47% for the neutrophil-to-lymphocyte ratio (NLR), 14.59% for the systemic inflammation response index (SIRI), and 11.13% for the systemic immune-inflammation index (SII). This negative relationship remained robust across subgroups and in the sensitivity analyses.

**Conclusion:**

An elevated DI-GM, which reflects a healthier microbiota state, was associated with a reduced prevalence of frailty. This relationship was partially mediated through inflammatory biomarkers.

## 1 Introduction

Population ageing is a global trend. It has been reported that by 2050, more than one-fifth of the global population will be older than 60 years. Although the average life expectancy has increased to 73.3 years, healthy life expectancy has not proportionally increased, and frailty has become a significant challenge for older adults (1). Frailty is a clinical syndrome that affects multiple systems and threatens the health of 12% of the elderly population (2). This condition is characterised by a decline in the ability to withstand external stressors, thus disrupting normal physiological homeostasis (3). Frailty not only increases the risks of hospitalisation, disability, and mortality among older individuals but also increases the economic burden of health care on society (4, 5). Although frailty is reversible (6) and interventions such as lifestyle modifications (including exercise and diet) and a comprehensive, multidisciplinary diagnostic and therapeutic approach are widely advocated, their effectiveness has yet to be firmly established (7). Therefore, further investigations into individualised protocols for reversing frailty are essential for addressing the health challenges experienced by older adults.

The gut microbiota is essential for human health and influences the regulation of the nervous system, immune function, and metabolic homeostasis (8). The composition of the gut microbiome changes throughout the lifespan. Specifically, it remains relatively stable between the ages of 3 and 65 years; however, as individuals become older, both the abundance and diversity of the gut microbiota decrease. This shift is associated with the development of a proinflammatory phenotype driven by factors such as diet, medication use, disease, and lifestyle changes (9, 10). Moreover, harmful microbiota and their undesirable metabolites can enter the systemic circulation through a compromised intestinal mucosal barrier, thereby leading to systemic inflammation and the progression of various diseases (11). Frailty is an age-related syndrome, and the role of the gut microbiota in the development of frailty has garnered increasing attention from the scientific community. Several studies involving large populations have demonstrated that, compared with nonfrail individuals, frail individuals exhibit a lower abundance of short-chain fatty acid (SCFA)-producing bacteria and reduced microbial diversity, accompanied by altered intestinal mucosal permeability (12). Moreover, it has been suggested that the gut microbiota not only participates in the pathological processes of frailty but also may represent a causal and genetic link between these two factors (13–17). Clinical studies have indicated that the rational use of prebiotics can significantly alleviate frailty symptoms (16), which is potentially achieved through the gut-muscle axis (18, 19). In aged mice undergoing faecal microbiota transplantation (FMT), improvements in mucosal barrier function and microbial composition were observed. These improvements were marked by an increase in the abundance of SCFA-producing bacteria and a reduction in circulating inflammatory markers, which collectively contributed to improved frailty symptoms (18). Therefore, the active maintenance of the gut microbial ecological balance (which consequently suppresses systemic chronic inflammation) may serve as an effective strategy for alleviating frailty.

Dietary factors play a pivotal role in the development and progression of frailty. A clinical study involving 612 frail and nonfrail older adults demonstrated that 12 months of adherence to the Mediterranean diet significantly improved the gut microbiota composition, thereby consequently reducing frailty risk and promoting healthy ageing. Supporting evidence from a cohort study of Israeli older adults revealed a significant inverse dose–response relationship between diet quality and frailty incidence. These findings are further corroborated by meta-analyses indicating that long-term Mediterranean diet adherence is associated with reduced frailty risk in ageing populations. Diet is a key modulator of the gut microbiome (20, 21). Recently, Kase et al. (22) developed a novel index for assessing gut health known as the dietary index for the gut microbiota (DI-GM), which is based on 14 foods or nutrients associated with gut ecological health. Current studies have indicated that the DI-GM is negatively associated with depression and age-related biological ageing, which is potentially mediated by body mass index (BMI) (23, 24).

Chronic inflammation is intimately involved in the pathological progression of frailty (25, 26). Accumulating evidence has demonstrated that, compared with their nonfrail counterparts, frail individuals exhibit significantly elevated levels of proinflammatory cytokines, particularly interleukin-6 and tumour necrosis factor-alpha (27, 28). The neutrophil-to-lymphocyte ratio (NLR), systemic inflammation response index (SIRI), and systemic immune-inflammation index (SII) are inflammatory biomarkers derived from routine haematological parameters. Among these biomarkers, the SII (which is a composite measure based on platelet, neutrophil, and lymphocyte counts) has been established as a reliable biomarker of systemic inflammation (29). Similarly, the SIRI is calculated using monocyte, neutrophil, and lymphocyte counts (30). These inflammatory indices have been extensively utilised to evaluate systemic inflammatory and immune responses, with substantial evidence linking them to the risk of frailty (31–33).

However, the relationship between the DI-GM and frailty status in older adults remains underexplored, with the mediating role of inflammatory biomarkers in this association being poorly understood. Given the established importance of gut microbiome homeostasis in frailty development and the well-documented contribution of systemic chronic inflammation, we conducted a population-based study using data from the National Health and Nutrition Examination Survey (NHANES). This study had two primary objectives: (1) to examine the associations between the DI-GM (representing microbiome health status as reflected by dietary intake) and frailty prevalence and (2) to investigate the mediating effects of specific inflammatory biomarkers, including the NLR, SIRI, and SII.

## 2 Methods

### 2.1 Data sources

The NHANES is conducted on a biennial basis and serves as a representative national survey, for which voluntary informed consent is received from all of the participants. The study recruited participants using a complex stratified sampling method and collected data on physical status, laboratory tests, and questionnaires from noninstitutionalised residents of the United States. After review by the National Center for Health Statistics Ethics Review Board, the NHANES was determined to be in full compliance with ethical standards.

### 2.2 Study population

**Figure 1:**
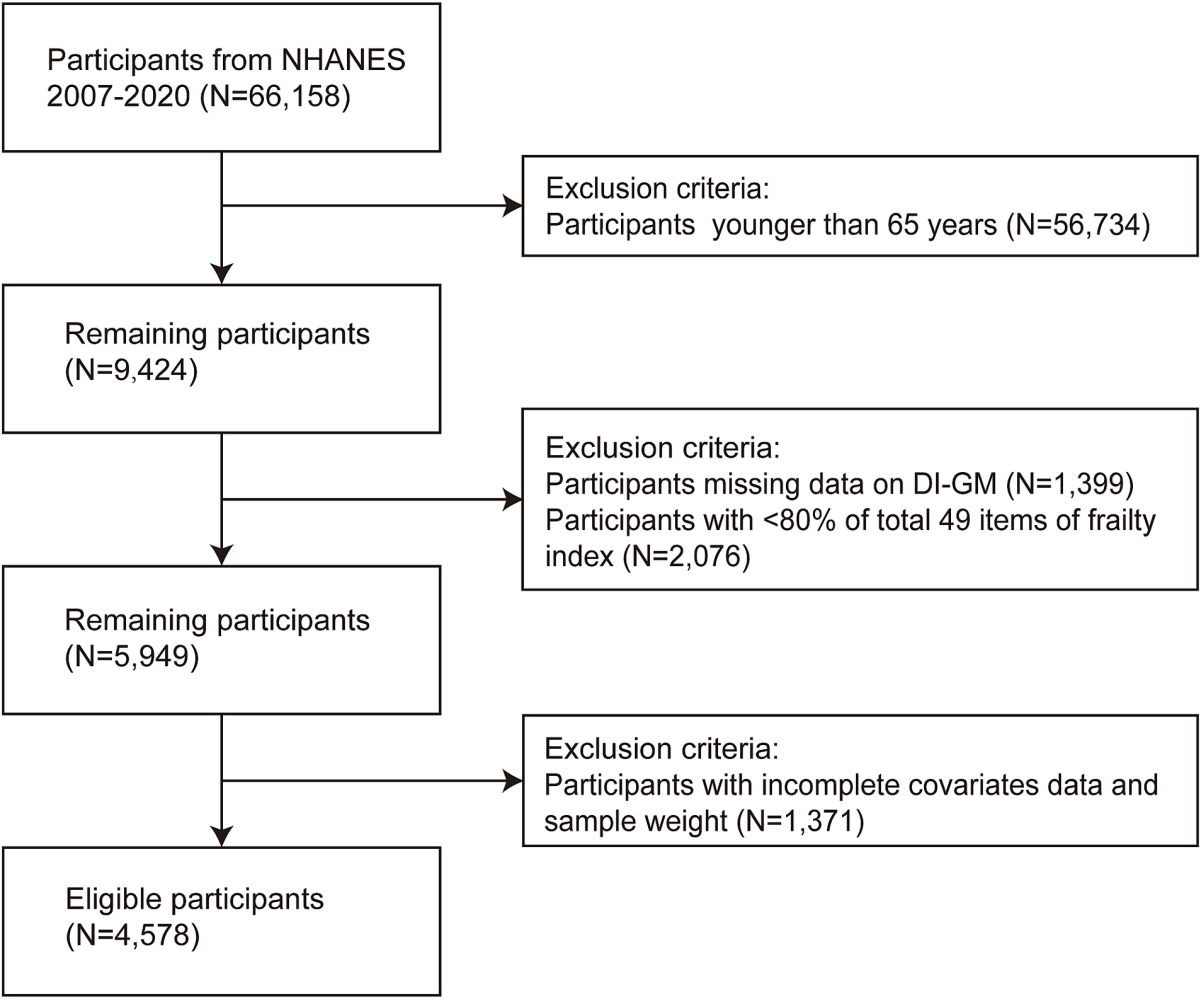
Flow chart of the participants exclusion process.

In the NHANES dataset, nutrient data relevant to the DI-GM assessment are available only for the years 2007–2020. Therefore, this study focused on the 66,148 participants recruited during this period. After applying a series of exclusion criteria, 4,578 eligible participants were retained for analysis, as shown in Figure 1.

### 2.3 Diagnosis of frailty

The outcome variables in this study were assessed using the frailty index (FI) created by Rockwood et al., which is the most widely used tool for diagnosing frailty (34). The FI assessment in this study included 49 health deficits encompassing a broad range of conditions, such as depression, cognitive impairment, comorbidities, and dependence. Importantly, a reliable FI assessment requires that no more than 20% of the health deficit items be missing. After a medical professional identifies health deficits, the ratio of the number of actual deficits to the total number of health deficit items yields the final FI score (reference range: 0–1) (Supplementary Table 1). According to previous studies, a diagnosis of frailty is made when the FI exceeds 0.21, and the FI value is directly proportional to the degree of frailty (35, 36).

### 2.4 Assessment of the dietary indices of the gut microbiota

As demonstrated in Supplementary Table 2, the DI-GM index was assessed based on 14 foods and nutrients, including 10 beneficial and 4 harmful components, all of which are associated with the gut microbiota profile. Specifically, foods that met the following three criteria were defined as beneficial: foods demonstrating increases in both *α*-diversity and *β*-diversity indices, foods demonstrating increases in total SCFAs, and foods demonstrating a balanced Firmicutes/Bacteroidetes ratio. All of the other components were classified as harmful. A score of 0 was assigned when the energy provided by fat exceeded 40% or when the intake of harmful components surpassed the sex-specific median; otherwise, a score of 1 was assigned. For the beneficial components, a score of 1 was assigned for intake above the sex-specific median, and 0 was assigned for intake below this median. Finally, the DI-GM score was determined by summing the scores of all 14 components, with higher scores indicating a healthier gut microbiota (reference range: 0–14) (22).

### 2.5 Assessment of mediating indicators

In this study, several distinct inflammatory biomarkers (including the NLR, SIRI, and SII) were evaluated for their mediating effects. Specifically, the NLR was calculated by dividing neutrophil counts by lymphocyte counts (37), whereas the SII was derived by multiplying platelet counts by the NLR (29). The SIRI was computed as the product of monocyte counts and neutrophil counts divided by lymphocyte counts (30). All of the blood samples for these measurements were collected by trained nursing staff and uniformly processed at a designated laboratory using an automated haematology analyser (Coulter DxH 800).

### 2.6 Covariates

We comprehensively considered potential covariates based on relevant studies. Specifically, these variables included sex (male or female), age (continuous), race (non-Hispanic White, non-Hispanic Black, Mexican American, other non-Hispanic, or other races), smoking status (current, former, or never), alcohol consumption (never, former, light, moderate, or heavy), total energy intake (continuous), physical activity (PA) (<600 MET-min/week or ≥600 MET-min/week), education level (less than high school, high school graduate, high school or higher), marital status (married/living with partner, never married, or widowed/divorced/separated), and poverty-to-income ratio (PIR) (<1.3, 1.3–3.5, or ≥3.5).

Those who had smoked more than 100 cigarettes during their lifetime were categorised as smokers. Current smokers were defined as individuals who still smoked, whereas former smokers were those individuals who had quit smoking. Alcohol consumption was assessed by asking whether individuals had consumed more than 12 alcoholic drinks in their lifetime and if they were currently drinking. The participants were categorised as never drinkers, former drinkers, or current drinkers. Among current drinkers, alcohol intake was classified into light, moderate, and heavy categories based on daily alcohol consumption. For women, light drinkers consumed less than 1 cup of alcohol per day, heavy drinkers consumed at least 3 cups per day, and moderate drinkers were those who consumed between these two amounts. For men, light drinkers consumed fewer than 2 cups per day, heavy drinkers consumed at least 4 cups per day, and moderate drinkers consumed between these two amounts. Additionally, participants who binged regarding alcohol intake more than 5 times per month were classified as heavy drinkers, whereas those who binged 2 to 5 times per month were categorised as moderate drinkers. Total energy intake was calculated as the average daily intake based on two 24-h dietary recall questionnaires. PA was assessed weekly via metabolic equivalents.

### 2.7 Statistical analysis

The NHANES uses a complex multistage stratified sampling method to recruit participants. In this study, all of the samples were weighted according to the officially recommended principle of minimum sample weight. As a result, the final statistical findings are generalisable to the entire U. S. population. We used *t*-tests and analysis of variance to compare differences in continuous variables between the participants. The chi-square test was applied to analyse differences in categorical variables. For continuous variables, the results of both comparisons are presented as the means (standard deviations), and for categorical variables, the results are presented as frequencies (proportions). Logistic regression models were developed to explore the potential relationships between the gut health status (as represented by the DI-GM) and frailty comprehensively. Linear regression was applied to examine the associations between the DI-GM and three inflammatory indicators (the NLR, SIRI, and SII). Three hierarchical models were constructed to examine the associations. Model 1 was an unadjusted crude model without covariates. Model 2 was partially adjusted for demographic factors, including race/ethnicity (non-Hispanic White, non-Hispanic Black, Mexican American, other Hispanic, and other races), age (continuous), and sex. Model 3 was fully adjusted with additional covariates: smoking status (current, former, never), education level (below high school, high school graduate, more than high school), PA level (<600 MET-min/week or ≥600 MET-min/week), alcohol consumption frequency (never, former, light, moderate, heavy), marital status (married/cohabiting, never married, widowed/divorced/separated), total energy intake (continuous), and PIR (<1.3, 1.3–3.5, ≥3.5). To assess the dose–response relationship, restricted cubic splines (RCSs) with three knots were fitted, adjusting for all potential confounders. The overall *p* value indicates the global association between DI-GM and frailty, whereas the nonlinear *p* value specifically tests for potential nonlinear trends. The ‘mediation’ package was used to evaluate the mediating effects of inflammatory biomarkers. Mediation analysis was performed with 1,000 bootstrap resamples and covariate adjustment to examine whether three inflammatory indicators (the NLR, SIRI, and SII) mediated the association between the DI-GM and frailty. The mediated proportion value was calculated using the following formula: indirect effect/(indirect effect + direct effect) × 100%. In this study, the direct effect represented the association between the DI-GM and frailty, whereas the indirect effect evaluated whether the DI-GM influenced frailty incidence via inflammatory biomarkers. Stratified analysis and sensitivity analysis were conducted to further confirm the consistency of the results. Subgroup analyses and interaction tests were conducted according to sex, age, race, smoking status, alcohol consumption, education level, hypertension status, and diabetes status, with adjustments being performed for all of the covariates except for the stratification variables. For the sensitivity analysis, we first adjusted for the NHANES cycle. Second, individuals with inappropriate energy intake (< 800 kcal/day or ≥ 4,200 kcal/day for males; < 500 kcal/day or ≥ 3,500 kcal/day for females) were also excluded from the study. In the third scenario, individuals younger than 70 years were not considered. All of the statistical analyses were performed using R software (version 4.4.2), with a *p* value < 0.05 indicating statistical significance.

## 3 Results

### 3.1 Analysis of baseline characteristics of the participants

Among the 4,578 eligible participants, the prevalence of frailty was 35.50%, with a mean DI-GM of 5.38 (0.04). The average age of the individuals was 72.79 years (0.14), with 2,302 females and 2,276 males included. Compared with nonfrail individuals, frail patients were older, included a lower proportion of non-Hispanic White participants, were less likely to be married or cohabiting, were more likely to be current smokers, exhibited lower levels of PA, had lower education and income levels, included a lower proportion of current drinkers, consumed less total energy, and demonstrated higher NLR, SIRI, and SII values. Additionally, frail individuals presented significantly lower DI-GM scores than their nonfrail counterparts did (Table 1).

**Table 1:** Weighted comparison of baseline characteristics.

Variables	Total (*N* = 4,578)	Non-frailty (*N* = 2,953)	Frailty (*N* = 1,625)	*p*-value
Age (years)	72.79 (0.14)	72.25 (0.15)	73.95 (0.23)	< 0.0001
Gender (%)				0.251
Female	2,302 (54.61)	1,431 (53.70)	871 (56.55)	
Male	2,276 (45.39)	1,522 (46.30)	754 (43.45)	
Race (%)				0.003
Mexican American	414 (3.42)	244 (2.77)	170 (4.79)	
Non-Hispanic Black	753 (7.01)	479 (6.68)	274 (7.71)	
Non-Hispanic White	2,787 (82.39)	1816 (83.80)	971 (79.40)	
Other Hispanic	387 (3.05)	247 (2.78)	140 (3.64)	
Other Race	237 (4.13)	167 (3.97)	70 (4.46)	
Education (%)				< 0.0001
Below high school	655 (8.03)	335 (5.87)	320 (12.64)	
High school graduate	1767 (35.16)	1,080 (32.22)	687 (41.42)	
Above high school	2,156 (56.81)	1,538 (61.91)	618 (45.94)	
Marital status (%)				< 0.0001
Married/Living with Partner	2,614 (62.41)	1825 (67.06)	789 (52.50)	
Widowed/Divorced/Separated	1787 (34.73)	1,017 (30.14)	770 (44.52)	
Never married	177 (2.86)	111 (2.80)	66 (2.98)	
PA (MET-mins/week)				< 0.0001
< 600	2,550 (50.57)	1,353 (41.19)	1,197 (70.60)	
≥ 600	2028 (49.43)	1,600 (58.81)	428 (29.40)	
PIR (%)				< 0.0001
< 1.3	1,276 (17.45)	659 (12.88)	617 (27.21)	
1.3–3.5	2048 (45.01)	1,322 (43.51)	726 (48.20)	
≥ 3.5	1,254 (37.54)	972 (43.61)	282 (24.59)	
Drinking status (%)				< 0.0001
Never	821 (15.34)	492 (14.64)	329 (16.85)	
Former	1,386 (26.41)	731 (21.55)	655 (36.78)	
Mild	1768 (45.03)	1,269 (48.63)	499 (37.34)	
Moderate	363 (9.07)	282 (10.67)	81 (5.64)	
Heavy	240 (4.15)	179 (4.51)	61 (3.38)	
Smoking status (%)				0.002
Never	2,193 (49.37)	1,470 (51.85)	723 (44.09)	
Former	1957 (43.10)	1,232 (41.60)	725 (46.31)	
Now	428 (7.52)	251 (6.55)	177 (9.60)	
Energy intake (kcal/day)	1826.03 (14.47)	1858.68 (17.11)	1756.39 (25.06)	0.001
NLR	2.56 (0.03)	2.40 (0.04)	2.90 (0.07)	< 0.0001
SIRI	1.55 (0.03)	1.41 (0.03)	1.85 (0.05)	< 0.0001
SII	580.55 (10.38)	542.23 (11.05)	662.29 (17.36)	< 0.0001
DI-GM (Continuous)	5.38 (0.04)	5.51 (0.05)	5.09 (0.06)	< 0.0001
DI-GM (Tertiles)				< 0.0001
T1 (≤4)	1,599 (31.58)	952 (28.90)	647 (37.30)	
T2 (5–6)	1882 (41.37)	1,219 (40.76)	663 (42.65)	
T3 (≥7)	1,097 (27.05)	782 (30.33)	315 (20.05)	

Comparisons of categorical variables using the chi-square test. Continuous variables were compared using *t*-test. PA, physical activity; MET, metabolic equivalents; PIR, poverty income ratio; NLR, neutrophil-to-lymphocyte ratio; SIRI, systemic inflammation response index; SII, systemic immunity-inflammation index; DI-GM, dietary index for gut microbiota.

Furthermore, the characteristics of the participants varied across different levels of the DI-GM. When the DI-GM was divided into tertiles, substantial differences were identified in race, sex, education background, PA, socioeconomic status, alcohol consumption, energy intake, smoking status, the NLR, the SIRI, and the SII. Furthermore, as the DI-GM level increased, the proportion of frail individuals significantly decreased (Supplementary Table 3).

### 3.2 Association of the DI-GM with frailty

To examine the association between DI-GM and frailty, we performed a multifactorial logistic regression analysis. For the continuous DI-GM, each unit increase in the DI-GM was significantly associated with a 12% reduction in the prevalence of frailty in Models 1 and 2 (Model 1: OR = 0.88, 95% CI: 0.83, 0.92, *p* < 0.0001; Model 2: OR = 0.88, 95% CI: 0.83, 0.93, *p* < 0.0001). After adjusting for all of the covariates, the negative relationship between the DI-GM and frailty remained unchanged (OR = 0.94, 95% CI: 0.89, 0.99, *p* = 0.020). When the DI-GM was divided into tertiles, participants in the highest tertile were significantly less likely to experience frailty than were those in the lowest tertile, with corresponding OR values of 0.51, 0.53, and 0.70 for Models 1, 2, and 3, respectively (Model 1: OR = 0.51, 95% CI: 0.41, 0.65, *p* < 0.0001; Model 2: OR = 0.53, 95% CI: 0.42, 0.68, *p* < 0.0001; Model 3: OR = 0.70, 95% CI: 0.53, 0.91, *p* = 0.008) (Table 2).

**Table 2:** Weighted logistic regression for association between DI-GM and the prevalence of frailty.

Exposures	Model 1 [OR (95% CI) *p*-value]	Model 2 [OR (95% CI) *p*-value]	Model 3 [OR (95% CI) *p*-value]
DI-GM (Continuous)	0.88 (0.83,0.92) < 0.0001	0.88 (0.83,0.93) < 0.0001	0.94 (0.89,0.99) 0.020
DI-GM (Tertiles)			
T1 (≤4)	ref	ref	ref
T2 (5–6)	0.81 (0.66,0.99) 0.044	0.81 (0.65,1.00) 0.050	0.89 (0.70,1.14) 0.357
T3 (≥7)	0.51 (0.41,0.65) < 0.0001	0.53 (0.42,0.68) < 0.0001	0.70 (0.53,0.91) 0.008
*P* for trend	<0.0001	<0.0001	0.008

Model 1: Adjusted for no variables. Model 2: Adjusted for race, gender, and age. Model 3: Adjusted for gender, age, race, PIR, education level, marital status, alcohol consumption, smoking status, PA, energy intake. DI-GM, dietary index for gut microbiota; OR, odds ratio.

**Figure 2:**
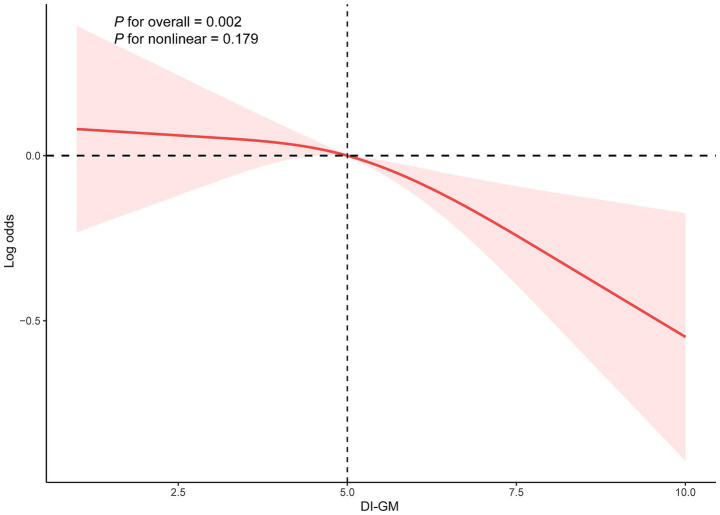
Restricted cubic spline fitting for the association between DI-GM and frailty. Adjusted for gender, age, race, PIR, education level, marital status, alcohol consumption, smoking status, PA, energy intake. DI-GM: dietary index for gut microbiota.

RCS analysis was employed to examine the dose–response relationship between DI-GM and the prevalence of frailty while assessing potential nonlinear associations. As illustrated in Figure 2, the RCS regression model with three knots demonstrated a significant negative linear association between DI-GM and the prevalence of frailty after adjustment for sex, age, race, PIR, education level, marital status, alcohol consumption, smoking status, PA, and energy intake (*P* for nonlinearity = 0.179).

### 3.3 Correlations between the DI-GM and inflammatory biomarkers

Linear regression analyses revealed significant associations between DI-GM and various inflammatory biomarkers. After full adjustment for all of the covariates, the DI-GM demonstrated inverse correlations with the NLR, SIRI, and SII (NLR: *β* = −0.05, 95% CI: −0.08, −0.02, *p* = 0.003; SIRI: *β* = −0.04, 95% CI: −0.06, −0.01, *p* = 0.008; SII: *β* = −12.28, 95% CI: −23.23, −1.33, *p* = 0.029). When the DI-GM was categorised into tertiles, participants in the highest tertile exhibited significantly lower NLR, SIRI, and SII values than did those in the lowest tertile (NLR: β = −0.26, 95% CI: −0.41, −0.11, *p* = 0.001; SIRI: β = −0.18, 95% CI: −0.30, −0.07, *p* = 0.003; SII: β = −60.07, 95% CI: −107.40, −12.74, *p* = 0.014) (Supplementary Table 4).

### 3.4 Mediation analysis

**Figure 3:**
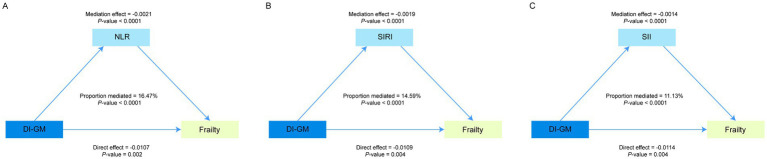
The mediating role of inflammatory biomarkers in the relationship between DI-GM and Frailty. Panels **(A–C)** illustrate the mediating effects of NLR, SIRI, and SII, respectively. Adjusted for gender, age, race, PIR, education level, marital status, alcohol consumption, smoking status, PA, energy intake. NLR, neutrophil-to-lymphocyte ratio; SIRI, systemic inflammation response index; SII, systemic immunity-inflammation index; DI-GM, dietary index for gut microbiota.

When considering the potential role of immune and inflammatory responses in the association between the gut microbiota and frailty, we performed mediation analyses to evaluate the mediating effects of multiple inflammatory biomarkers, including the NLR, SIRI, and SII. As shown in Figures 3A–C, these inflammatory markers collectively partially mediated the relationship between the DI-GM and frailty. Specifically, the mediation proportions were 16.47% for the NLR, 14.59% for the SIRI, and 11.13% for the SII (*p* < 0.05).

### 3.5 Subgroup analyses

**Figure 4:**
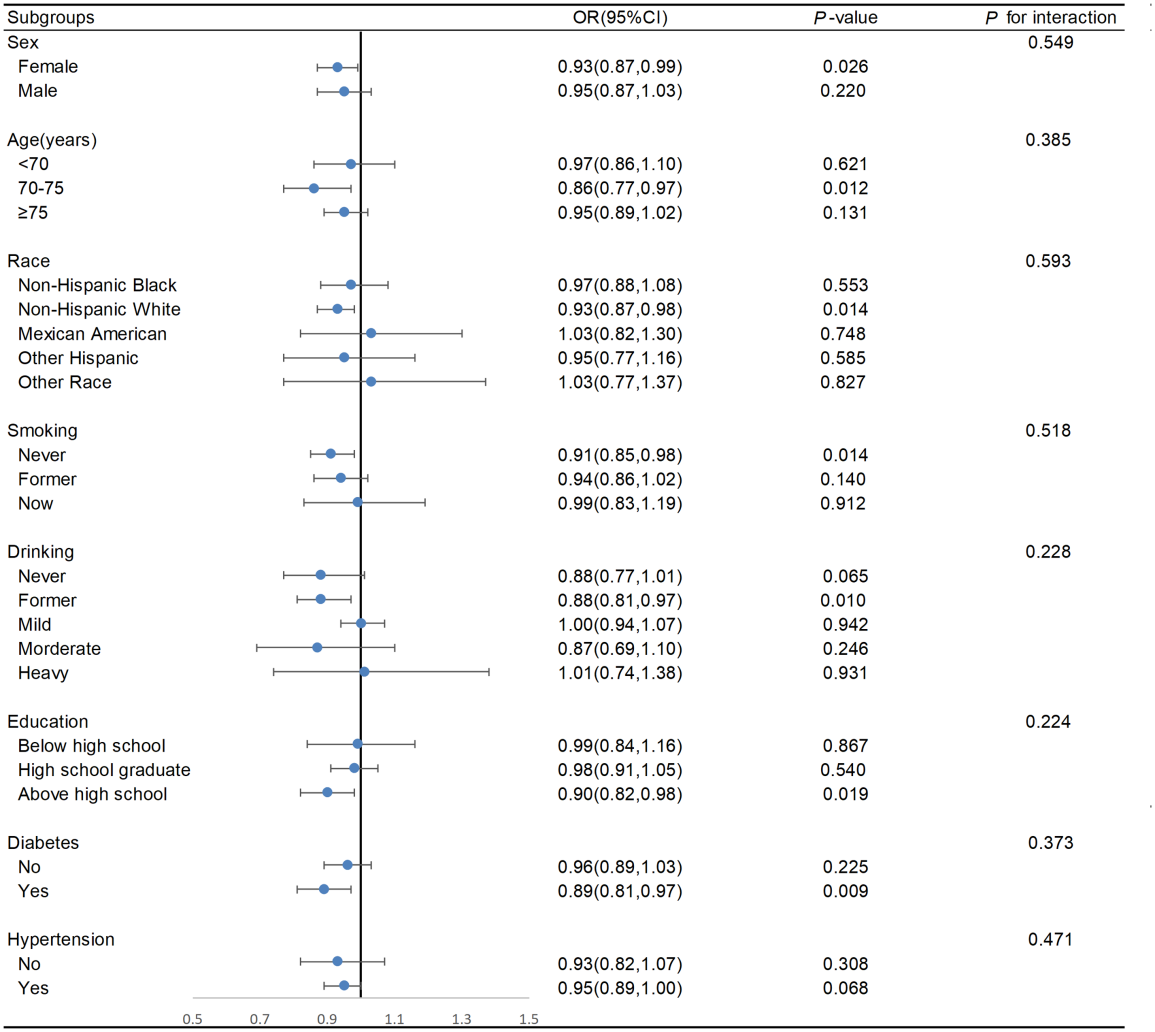
Subgroup analysis of the association between DI-GM and frailty. Adjusted for gender, age, race, PIR, education level, marital status, alcohol consumption, smoking status, PA, energy intake, except the subgroup factors themselves. DI-GM, dietary index for gut microbiota; OR, odds ratio.

Individuals were categorised into different subgroups for stratified analysis to examine the interaction of these covariates with the DI-GM. As shown in Figure 4, race, age, sex, alcohol consumption, education background, smoking status, diabetes status, and hypertension status did not significantly interact with the DI-GM (*P* for interaction > 0.05), suggesting that these factors do not influence the negative association between the DI-GM and frailty.

### 3.6 Sensitivity analysis

We performed various sensitivity analyses to examine the accuracy of this relationship. In Model 3, further adjustment for the NHANES cycles still revealed a negative relationship between the DI-GM and frailty (Supplementary Table 5). Participants with inappropriate energy intake were subsequently excluded. According to the three logistic regression models, a higher DI-GM value was associated with a lower prevalence of frailty, which aligns with the main findings of the present study (Supplementary Table 6). In the third analysis, individuals aged less than 70 years were excluded. The results revealed that both continuous and tertile-based DI-GM values were similarly associated with frailty, which was in accordance with the previously determined findings (Supplementary Table 7).

## 4 Discussion

The DI-GM is a novel indicator for evaluating the health status of the gut microbiota. This cross-sectional study explored (for the first time) the association between the DI-GM and frailty. Overall, the nonfrail population presented a healthier gut microbiome than did the frail population. A dose–response relationship was indicated between the DI-GM and the prevalence of frailty across all three of the developed models, and this association remained consistent across different populations. Several sensitivity analyses further supported these findings. Mediation analysis indicated that DI-GM reduced the likelihood of frailty, which was partially achieved by mediating the expression of inflammatory biomarkers. These findings demonstrate that the DI-GM (via the assessment of dietary intake to reflect an individual’s gut microbiota health) is promising as a biomarker for the early identification of populations at high risk for frailty. Importantly, the optimisation of dietary patterns to improve DI-GM scores may enhance gut microbial homeostasis, thereby offering significant clinical value in guiding personalised therapeutic strategies for elderly patients with frailty.

The gut microbiota is a critical component in maintaining host health throughout the life cycle. The gut microbial environment undergoes dynamic changes as individuals age. Microbes typically begin to colonise the gut during foetal life and gradually diversify beginning at approximately 3 years of age, after which they remain relatively stable during adulthood. However, with advancing age, inflammatory changes occur in the gut due to increased exposure to external stressors, organ decline, shifts in dietary patterns, and poor lifestyle choices (9, 10). These changes are accompanied by an increase in microbial variability and a decrease in microbial diversity. The microbial composition is subsequently characterised by an increased proportion of opportunistic pathogens and a reduced number of immune-modulating commensal microorganisms, which are often derived from Firmicutes. Damage to the intestinal mucosal barrier further promotes the diffusion of inflammatory factors and harmful metabolites into the circulatory system, thereby triggering a widespread chronic inflammatory response that accelerates the progression of age-related diseases (38–40). A study analysing the gut microbiome and its metabolites in 178 elderly individuals of Irish descent revealed that microbiota-associated ageing was associated with poorer psychological and health scores, compromised nutritional status, and elevated levels of inflammatory markers, all of which accelerate unhealthy ageing (41). Moreover, the remodelling of the microbial environment in naturally ageing mice via FMT effectively mitigated signs and symptoms of systemic ageing, including decreased glucose tolerance, reduced antioxidant capacity, liver dysfunction, and chronic inflammation (42, 43). Clinical research has also demonstrated that probiotic supplementation can increase bone health and cognitive function in older adults (44–46). Collectively, these results underscore the significant role of gut microbial ageing in the development of age-related diseases and the progression of unhealthy ageing.

Frailty, which is a common age-related syndrome with a prevalence of up to 12% in elderly individuals, represents a major challenge to human health and longevity (2). The strong association between the gut microbiota and ageing indicates that the gut microbiome could be a key factor in the development of frailty. Recently, multiple population-centred studies have highlighted the potential link between gut microbiota health and frailty in older adults (11, 13, 47–49). A study involving 1,693 community-dwelling older adults revealed that the gut microbiota of elderly individuals is predominantly composed of the phyla Firmicutes, Bacteroidetes, Proteobacteria, and Actinobacteria. Changes in frailty levels are associated with alterations in microbial composition and abundance, such as an increase in Bacteroides and decreases in Bifidobacterium and Lactobacillus, along with corresponding shifts in metabolic pathways. Research has demonstrated that after 12 weeks of intervention with a probiotic mixture, frailty symptoms exhibit partial improvements, as evidenced by increased walking speed and grip strength. Additionally, globulin levels are elevated, and the levels of certain inflammatory markers tend to decrease (16). A study by Pu et al. revealed an association between 18 microbial species and the degree of frailty, with 13 harmful genera increasing in relative abundance as frailty progressed, whereas the remaining 5 beneficial genera decreased in abundance. Mediation analysis indicated that this association was partially mediated via systemic inflammation, abnormal energy metabolism, and renal dysfunction. Furthermore, researchers have screened for microbes and metabolites associated with mortality and established a microbial FI, which demonstrated a strong association with 2-year mortality (15). A meta-analysis of 11 clinical studies revealed that, compared with healthy older adults, frail older adults presented reduced gut microbiota diversity and altered abundances of specific genera, which was characterised by a lower prevalence of Firmicutes and a reduced number of SCFA producers. Additionally, increased expression of serum zonulin was observed, thereby leading to intestinal permeability (leaky gut), along with increased levels of proinflammatory cytokines (12). To further investigate the genetic correlation and causality between the gut microbiota and frailty, Cui et al. conducted a genome-wide association study. The results revealed that Christensenellaceae R-7 was genetically associated with frailty and that 12 bacterial genera (including *Clostridium innocuum* and *Eubacterium coprostanoligenes*) could be involved in the development of frailty (47). However, Zhu et al. treated aged mice with FMT for 6 weeks, which resulted in significant improvements in grip strength and running speed, increased motor coordination and endurance, improved cognitive function, and reduced frailty scores. These findings suggest FMT as a promising approach for managing age-related frailty (18). Collectively, these studies underscore the potential role of gut microbiota homeostasis in delaying the onset and progression of frailty, which is consistent with our findings. Our results demonstrate that higher DI-GM scores, which are indicative of healthier gut microbial profiles, are significantly associated with reduced frailty prevalence.

Diet is a key regulator of gut microbial health (50, 51). Therefore, the modification of suboptimal dietary patterns to maintain microbial homeostasis is crucial for preventing frailty. Given the strong relationship observed between dietary habits and the gut microbiota, Kase et al. (22) conducted a comprehensive review of 106 studies and innovatively developed a novel index to assess the health of the gut microbiota. This index was developed based on a comprehensive assessment of the consumption of 14 foods or nutrients that are known to impact the gut microbiota (22). Currently, the DI-GM has been shown to reflect the health of the gut microbiota in relation to depression and biological age, with this association being partly mediated by weight loss (23, 24). Given the critical impact of the gut microbiota on the progression of frailty and ageing, the relationship between the DI-GM and frailty warrants further investigation. This study provides the first evidence of an inverse association between the DI-GM and frailty, thereby establishing this innovative metric as a promising biomarker for identifying high-risk populations susceptible to frailty. A study involving 612 elderly individuals from five countries also supported the notion that dietary adjustments can decrease the risk of frailty and promote healthier ageing. Specifically, following a 12-month intervention with a Mediterranean diet, participants exhibited changes in their gut microbiota composition, which were associated with reduced frailty, improved cognitive function, and lower levels of inflammatory markers (52). Moreover, a study of Chinese patients with lung cancer revealed that poor dietary quality and inadequate nutrient intake contribute to frailty development via alterations in the gut microbiota, which were characterised by an increased relative abundance of Actinobacteria and reduced *β* diversity (53). These findings are consistent with our results, thus suggesting that dietary modulation to promote gut microbial health represents a promising intervention strategy for reversing frailty in older adults.

The precise mechanisms by which dietary factors improve frailty via the modulation of gut microbiota health remain underexplored but may involve the following pathways. Different dietary patterns substantially influence an individual’s gut microbial composition. Populations adhering to plant-based diets typically present reduced abundances of opportunistic pathogens such as Firmicutes and Proteobacteria, along with increased levels of beneficial bacteria, including Bacteroidetes and Bifidobacterium (50, 51). Conversely, Western dietary patterns characterised by high sugar, high fat, and low fibre intake tend to promote the proliferation of opportunistic pathogens while diminishing beneficial bacterial populations (50, 51). Additionally, the gut microbiota and its metabolites participate in frailty progression via multiple mechanisms. For example, SCFAs (the end metabolites of dietary fibre fermented by gut microbes) are significantly reduced in older, frail populations (48). SCFAs help maintain glycolipid metabolism homeostasis in skeletal muscle, increasing mitochondrial resistance to oxidative stress and increasing the number of skeletal muscle fibres, thereby reducing fat accumulation and preventing muscle mass loss (54–56). Additionally, SCFAs are important inhibitors of the inflammatory response; moreover, they also enhance the protective function of the intestinal epithelium by promoting the expression of mucin 2 and tight junction proteins (57). Lipopolysaccharide, which is a critical metabolic by-product of the gut microbiota, accumulates in the systemic circulation in an age-dependent manner. It enters the circulation through a leaky gut, attaches to toll-like receptor 4 on innate immune cells, and activates the downstream protein NF-κB, thereby triggering a systemic inflammatory response (18, 58). This response increases muscle protein degradation, inhibits muscle fibre regeneration, and ultimately leads to reduced muscle mass and frailty (59). Additionally, dietary choline and L-carnitine are metabolised by the gut microbiota into trimethylamine, which is subsequently oxidised by hepatic flavin monooxygenases to form trimethylamine N-oxide (TMAO) (60). TMAO disrupts the tight junctions of intestinal epithelial cells by decreasing the expression of the claudin-1 protein, thereby affecting the structure of the microbiota. This alteration is characterised by an abnormal ratio of the phylum Firmicutes to the phylum Bacteroidetes, which is accompanied by a decreased adaptability of intestinal microorganisms to external stimuli, thus ultimately leading to increased vulnerability of the organism (61). Additionally, TMAO may interfere with glucose metabolism and stimulate an inflammatory response, thereby affecting muscle mass and immune function, both of which are critical factors in the development of frailty (62, 63). In conclusion, the gut microbiota may influence frailty progression via the modulation of energy metabolism, chronic inflammatory responses, and intestinal barrier dysfunction. Consistent with previous research, our mediation analysis further revealed that the inverse association observed between DI-GM-represented gut microbial health and frailty is partially mediated by inflammatory pathways.

This study identifies (for the first time) a negative relationship between the DI-GM and frailty. This research focused on elucidating the notion that an increase in the intake of beneficial foods or nutrients (which would correspondingly improve the gut microbial environment) may help reduce the risk of frailty. These innovative results provide a theoretical foundation for the development of preventive and therapeutic measures targeting frailty in elderly individuals. Additionally, this study is based on the NHANES, which is a large-scale dataset representative of the entire U. S. population, thereby lending reliability and generalisability to our results. Finally, we tested the robustness of the findings via stratified analyses.

Despite these strengths, there are several limitations to consider. First, this was a cross-sectional study that did not dynamically monitor changes in frailty status in response to dietary modifications. As a result, we are unable to establish a causal relationship between the DI-GM and frailty. Moreover, the NHANES did not assess the true gut microbial composition of the participants. Consequently, extensive cohort research is needed to further validate the effectiveness of DI-GM evaluation of gut microbiome health in predicting and reversing the progression of frailty. Second, the DI-GM was assessed via a 24-h dietary recall interview, which may have introduced memory bias into the results. Third, although we thoroughly considered demographic and lifestyle factors, there may be additional unobserved confounders that could influence the relationship between the DI-GM and frailty.

## 5 Conclusion

In summary, this study reveals a potential negative relationship between the DI-GM and frailty while highlighting the mediating role of inflammatory responses in this relationship. These findings not only support the potential use of the DI-GM as a biological marker for the early identification of high-risk populations for frailty but also provide a theoretical foundation for the development of targeted therapeutic interventions. From a clinical perspective, the optimisation of dietary patterns to improve diet quality may help maintain a balanced and functionally competent gut microbiome, thereby attenuating chronic systemic inflammation and potentially ameliorating frailty in older adults.

However, several critical research gaps remain to be addressed. First, large-scale prospective cohort studies are warranted to elucidate how specific gut microbial compositional changes influence frailty progression. Second, mechanistic studies are needed to delineate the precise pathways through which the gut microbiota modulates inflammatory processes to affect frailty development.

## Data Availability

The datasets presented in this study can be found in online repositories. The names of the repository/repositories and accession number(s) can be found at: http://www.cdc.gov/nchs/nhanes/.
