# Supplementary material for: The association between a dietary index for the gut microbiota and frailty in older adults: emphasising the mediating role of inflammatory indicators
Source: Front Nutr. 2025 Jul 9;12:1562278. doi: 10.3389/fnut.2025.1562278 (PMC12284950; doi:10.3389/fnut.2025.1562278)
Supplement: Supplementary file 1 [file Table_1.docx]

**Supplementary Table 1. Variables and scorings in the 49-Item Frailty Index.**

| **Items** | **scores** |
| --- | --- |
| **Cognition** |  |
| 1.experience confusion/memory problems | yes=1, no=0 |
| **Dependence** |  |
| 2.managing money difficulty | no difficulty=0, Some difficulty=0.33, much difficulty=0.66, unable to do=1 |
| 3.walking for a quarter mile difficulty | no difficulty=0, Some difficulty=0.33, much difficulty=0.66, unable to do=1 |
| 4.walking up ten steps difficulty | no difficulty=0, Some difficulty=0.33, much difficulty=0.66, unable to do=1 |
| 5.stooping, crouching, kneeling difficulty | no difficulty=0, Some difficulty=0.33, much difficulty=0.66, unable to do=1 |
| 6.lifting or carrying difficulty | no difficulty=0, Some difficulty=0.33, much difficulty=0.66, unable to do=1 |
| 7.house chore difficulty | no difficulty=0, Some difficulty=0.33, much difficulty=0.66, unable to do=1 |
| 8.preparing meals difficulty | no difficulty=0, Some difficulty=0.33, much difficulty=0.66, unable to do=1 |
| 9.standing up from armless chair difficulty | no difficulty=0, Some difficulty=0.33, much difficulty=0.66, unable to do=1 |
| 10.getting in and out of bed difficulty | no difficulty=0, Some difficulty=0.33, much difficulty=0.66, unable to do=1 |
| 11.using fork, knife, drinking from cup difficulty | no difficulty=0, Some difficulty=0.33, much difficulty=0.66, unable to do=1 |
| 12.dressing yourself difficulty | no difficulty=0, Some difficulty=0.33, much difficulty=0.66, unable to do=1 |
| 13.standing for long periods difficulty | no difficulty=0, Some difficulty=0.33, much difficulty=0.66, unable to do=1 |
| 14.grasp/holding small objects difficulty | no difficulty=0, Some difficulty=0.33, much difficulty=0.66, unable to do=1 |
| 15.attending social event difficulty | no difficulty=0, Some difficulty=0.33, much difficulty=0.66, unable to do=1 |
| 16.leisure activity at home difficulty | no difficulty=0, Some difficulty=0.33, much difficulty=0.66, unable to do=1 |
| 17.push or pull large objects difficulty | no difficulty=0, Some difficulty=0.33, much difficulty=0.66, unable to do=1 |
| **Depressive Conditions** |  |
| 18.have little interest in doing things | nearly every day = 1, more than half the days = 0.66, several days = 0.33, no =0 |
| 19.feeling down, depressed, or hopeless | nearly every day = 1, more than half the days = 0.66, several days = 0.33, no =0 |
| 20.trouble sleeping or sleeping too much | nearly every day = 1, more than half the days = 0.66, several days = 0.33, no =0 |
| 21.feeling tired or having little energy | nearly every day = 1, more than half the days = 0.66, several days = 0.33, no =0 |
| 22.poor appetite or overeating | nearly every day = 1, more than half the days = 0.66, several days = 0.33, no =0 |
| 23.feeling bad about yourself | nearly every day = 1, more than half the days = 0.66, several days = 0.33, no =0 |
| 24.trouble concentrating on things | nearly every day = 1, more than half the days = 0.66, several days = 0.33, no =0 |
| **Comorbidities** |  |
| 25.doctor ever said you had arthritis | yes = 1, no = 0 |
| 26.ever told you had thyroid problem | yes = 1, no = 0 |
| 27.ever told you had chronic bronchitis | yes = 1, no = 0 |
| 28.ever told you had cancer or malignancy | yes = 1, no = 0 |
| 29.ever told had congestive heart failure | yes = 1, no = 0 |
| 30.ever told you had coronary heart disease | yes = 1, no = 0 |
| 31.ever told you had angina/angina pectoris | yes = 1, no = 0 |
| 32.ever told you had heart attack | yes = 1, no = 0 |
| 33.ever told you had a stroke | yes = 1, no = 0 |
| 34.ever told you had high blood pressure | yes = 1, no = 0 |
| 35.doctor told you have diabetes | yes = 1, borderline=0.5, no =0 |
| 36.ever told you had weak/failing kidneys | yes = 1, no =0 |
| 37.urine leakage bother you? | greatly = 1, very much =0.75, somewhat= 0.5, only a little = 0.25, no=0 |
| **Hospital and Care** |  |
| 38.general health condition | excellent, very good, good = 0, fair, poor = 1 |
| 39.health now compared with 1 year ago | worse =1, better = 0 |
| 40.overnight hospital patient in last year | yes =1, no =0 |
| 41.times receive healthcare over past year | no=0, 1-4=0.5, ≥5 =1 |
| 42.number of prescription medicines taken | no =0, 1-4=0.5, ≥5 =1 |
| **Physical Anthropometry** |  |
| 43.body mass index (kg/m^2^) | <18.5, ≥30=1  ≥25, <30=0.5  ≥18.5,＜25=0 |
| **Laboratory values** |  |
| 44.glycohemoglobin (%) | 0%-5.7%=0, >5.7%=1 |
| 45.red blood cell count (million cells/ul) | M: ≥4.7, <6.1=0, Other=1  F: ≥4.2,＜5.4=0, Other =1 |
| 46.hemoglobin (g/dl) | M: ≥13.5, <18 =0, Other =1  F: ≥12, <16 =0, Other=1 |
| 47.red cell distribution width (%) | ≥11.6, <14.6=0, Other=1 |
| 48.lymphocyte percent (%) | ≥20, <40=0, Other=1 |
| 49.segmented neutrophils percent (%) | ≥40, <80=0, Other=1 |

**Supplementary Table 2. Evaluation Criteria for DI-GM。**

| **Components** | **Scoring criteria** |
| --- | --- |
| **Beneficial to gut microbiota** | |
| Avocados | Score 1: Consumption ≥ sex-specific median  Score 0: Otherwise |
| Broccoli |  |
| Chickpeas |  |
| Coffee |  |
| Cranberries |  |
| Fermented dairy (including yogurt, cheese, kefir, sour cream, buttermilk) |  |
| Fiber |  |
| Soybean (including Soy milk, Tofu) |  |
| Whole grains |  |
| **Unfavorable to gut microbiota** | |
| Refined grains | Score 1: Consumption ≥ sex-specific median  Score 0: Otherwise |
| Processed meat |  |
| Red meat |  |
| High-fat diet (% energy) | Score 0: Consumption≥40%  Score 1: Otherwise |

DI-GM: dietary index for gut microbiota.

**Supplementary Table 3. Comparison of baseline characteristics of participants with different DI-GM levels.**

| Variables | Total (N=4,578) | DI-GM | | | *P*-value |
| --- | --- | --- | --- | --- | --- |
|  |  | T1 (≤4) | T2 (5-6) | T3 (≥7) |  |
| Age (years) | 72.79(0.14) | 72.91(0.21) | 73.00(0.24) | 72.34(0.26) | 0.110 |
| Gender (%) |  |  |  |  | 0.027 |
| Female | 2302(54.61) | 729(50.65) | 984(56.72) | 589(56.02) |  |
| Male | 2276(45.39) | 870(49.35) | 898(43.28) | 508(43.98) |  |
| Race (%) |  |  |  |  | < 0.0001 |
| Mexican American | 414(3.42) | 163(4.22) | 168(3.41) | 83(2.49) |  |
| Non-Hispanic Black | 753(7.01) | 319(9.56) | 307(6.86) | 127(4.25) |  |
| Non-Hispanic White | 2787(82.39) | 888(78.52) | 1151(82.58) | 748(86.63) |  |
| Other Hispanic | 387(3.05) | 152(3.78) | 159(2.74) | 76(2.68) |  |
| Other Race | 237(4.13) | 77(3.93) | 97(4.40) | 63(3.95) |  |
| Education (%) |  |  |  |  | < 0.0001 |
| Below high school | 655(8.03) | 280(11.52) | 277(7.83) | 98(4.27) |  |
| High school graduate | 1767(35.16) | 687(39.89) | 712(35.42) | 368(29.22) |  |
| Above high school | 2156(56.81) | 632(48.59) | 893(56.75) | 631(66.51) |  |
| Marital status |  |  |  |  | 0.402 |
| Married/Living with Partner | 2614(62.41) | 887(59.82) | 1075(63.52) | 652(63.75) |  |
| Widowed/Divorced/Separated | 1787(34.73) | 641(36.73) | 748(34.19) | 398(33.22) |  |
| Never married | 177(2.86) | 71(3.46) | 59(2.29) | 47(3.02) |  |
| PA (MET-mins/week) |  |  |  |  | < 0.0001 |
| <600 | 2550(50.57) | 987(57.90) | 1049(52.68) | 514(38.80) |  |
| ≥600 | 2028(49.43) | 612(42.10) | 833(47.32) | 583(61.20) |  |
| PIR (%) |  |  |  |  | < 0.0001 |
| <1.3 | 1276(17.45) | 514(23.12) | 530(17.01) | 232(11.51) |  |
| 1.3-3.5 | 2048(45.01) | 747(46.68) | 830(44.87) | 471(43.26) |  |
| ≥3.5 | 1254(37.54) | 338(30.20) | 522(38.12) | 394(45.23) |  |
| Drinking status (%) |  |  |  |  | < 0.0001 |
| Never | 821(15.34) | 275(16.04) | 356(15.61) | 190(14.12) |  |
| Former | 1386(26.41) | 532(28.04) | 590(29.23) | 264(20.20) |  |
| Mild | 1768(45.03) | 557(41.42) | 700(42.45) | 511(53.18) |  |
| Moderate | 363(9.07) | 132(8.39) | 136(8.98) | 95(10.00) |  |
| Heavy | 240(4.15) | 103(6.11) | 100(3.73) | 37(2.49) |  |
| Smoking status (%) |  |  |  |  | 0.002 |
| Never | 2193(49.37) | 707(46.27) | 931(50.88) | 555(50.68) |  |
| Former | 1957(43.10) | 701(42.94) | 785(42.47) | 471(44.27) |  |
| Now | 428(7.52) | 191(10.79) | 166(6.65) | 71(5.05) |  |
| Energy intake (kcal/day) | 1826.03(14.47) | 1727.40(20.84) | 1828.50(22.51) | 1937.40(27.96) | < 0.0001 |
| NLR | 2.56(0.03) | 2.69(0.06) | 2.55(0.05) | 2.41(0.06) | 0.004 |
| SIRI | 1.55(0.03) | 1.62(0.04) | 1.58(0.04) | 1.40(0.04) | < 0.001 |
| SII | 580.55(10.38) | 603.46(18.52) | 588.84(14.72) | 541.13(14.49) | 0.014 |
| Frailty (%) |  |  |  |  | < 0.0001 |
| No | 2953(68.08) | 952(62.30) | 1219(67.09) | 782(76.35) |  |
| Yes | 1625(31.92) | 647(37.70) | 663(32.91) | 315(23.65) |  |

DI-GM: dietary index for gut microbiota; PA: physical activity; MET: metabolic equivalent; PIR: poverty income ratio; NLR: neutrophil-to-lymphocyte ratio; SIRI: systemic inflammation response index; SII: systemic immune-inflammatory index.

**Supplementary Table 4. Weighted linear regression for association between DI-GM and** **inflammatory biomarkers.**

| Exposures | Model1  [β (95% CI) *P*-value] | Model2  [β (95% CI) *P*-value] | Model3  [β (95% CI) *P*-value] |
| --- | --- | --- | --- |
| NLR | | | |
| DI-GM (Continuous) | -0.05(-0.09,-0.02) 0.001 | -0.05(-0.09,-0.02) 0.001 | -0.05(-0.08,-0.02) 0.003 |
| DI-GM (Tertiles) |  |  |  |
| T1 (≤4) | ref | ref | ref |
| T2 (5-6) | -0.13(-0.27, 0.00) 0.046 | -0.13(-0.26, 0.00) 0.051 | -0.12(-0.25, 0.01) 0.061 |
| T3 (≥7) | -0.27(-0.43,-0.12) <0.001 | -0.27(-0.42,-0.12) <0.001 | -0.26(-0.41,-0.11) 0.001 |
| *P* for trend | <0.001 | <0.001 | 0.001 |
| SIRI | | | |
| DI-GM (Continuous) | -0.04(-0.07,-0.02) <0.001 | -0.04(-0.07,-0.02) <0.001 | -0.04(-0.06,-0.01) 0.008 |
| DI-GM (Tertiles) |  |  |  |
| T1 (≤4) | ref | ref | ref |
| T2 (5-6) | -0.04(-0.14, 0.07) 0.455 | -0.03(-0.13, 0.07) 0.532 | -0.02(-0.12, 0.08) 0.740 |
| T3 (≥7) | -0.22(-0.33,-0.11) <0.001 | -0.22(-0.32,-0.11) <0.001 | -0.18(-0.30,-0.07) 0.003 |
| *P* for trend | <0.001 | <0.001 | 0.003 |
| SII | | | |
| DI-GM (Continuous) | -12.88(-22.98,-2.77) 0.013 | -14.00(-24.26, -3.73) 0.008 | -12.28(-23.23, -1.33) 0.029 |
| DI-GM (Tertiles) |  |  |  |
| T1 (≤4) | ref | ref | ref |
| T2 (5-6) | -14.62(-57.15, 27.91) 0.496 | -16.95(-59.53, 25.63) 0.430 | -14.21(-58.58, 30.17) 0.524 |
| T3 (≥7) | -62.33(-106.76,-17.90) 0.007 | -67.88(-112.24,-23.52) 0.003 | -60.07(-107.40,-12.74) 0.014 |
| *P* for trend | 0.008 | 0.004 | 0.015 |

Model 1: Adjusted for no variables.

Model 2: Adjusted for race, gender, and age.

Model 3: Adjusted for gender, age, race, PIR, education level, marital status, alcohol consumption, smoking status, PA, energy intake.

NLR: neutrophil-to-lymphocyte ratio; SIRI: systemic inflammation response index; SII: systemic immunity-inflammation index; DI-GM: dietary index for gut microbiota.

**Supplementary Table 5. Weighted logistic regression for association between DI-GM and the prevalence of frailty (adjusted for NHANES cycle)。**

| Exposures | [OR (95% CI) *P*-value] |
| --- | --- |
| DI-GM (Continuous) | 0.94(0.89,1.00) 0.034 |
| DI-GM (Tertiles) |  |
| T1 (≤4) | ref |
| T2 (5-6) | 0.89(0.70,1.14) 0.364 |
| T3 (≥7) | 0.71(0.54,0.93) 0.015 |
| *P* for trend | 0.015 |

Adjusted for gender, age, race, PIR, education level, marital status, alcohol consumption, smoking status, PA, energy intake, NHANES cycle.

DI-GM: dietary index for gut microbiota; OR: odds ratio.

**Supplementary Table 6. Weighted logistic regression for association between DI-GM and the prevalence of frailty (excluding inappropriate energy intake)。**

| Exposures | Model1 [OR (95% CI) *P*-value] | Model2 [OR (95% CI) *P*-value] | Model3 [OR (95% CI) *P*-value] |
| --- | --- | --- | --- |
| DI-GM (Continuous) | 0.88(0.84,0.92) <0.0001 | 0.88(0.84,0.93) <0.0001 | 0.94(0.89,0.99) 0.024 |
| DI-GM (Tertiles) |  |  |  |
| T1 (≤4) | ref | ref | ref |
| T2 (5-6) | 0.82(0.66,1.01) 0.060 | 0.81(0.65,1.02) 0.069 | 0.90(0.70,1.15) 0.391 |
| T3 (≥7) | 0.52(0.41,0.66) <0.0001 | 0.54(0.42,0.69) <0.0001 | 0.70(0.54,0.91) 0.009 |
| *P* for trend | < 0.0001 | < 0.0001 | 0.009 |

This model excludes participates with inappropriate energy intake(<500 kcal/day or ≥3,500 kcal/day for females, or <800 kcal/day or ≥4,200 kcal/day for males).

Model 1: Adjusted for no variables.

Model 2: Adjusted for race, gender, and age.

Model 3: Adjusted for gender, age, race, PIR, education level, marital status, alcohol consumption, smoking status, PA, energy intake.

DI-GM: dietary index for gut microbiota; OR: odds ratio.

**Supplementary Table 7. Weighted logistic regression for association between DI-GM and the prevalence of frailty (excluding participants less than 70 years of age)。**

| Exposures | Model1 [OR (95% CI) *P*-value] | Model2 [OR (95% CI) *P*-value] | Model3 [OR (95% CI) *P*-value] |
| --- | --- | --- | --- |
| DI-GM (Continuous) | 0.86(0.82,0.91) <0.0001 | 0.87(0.82,0.92) <0.0001 | 0.92(0.87,0.97) 0.004 |
| DI-GM (Tertiles) |  |  |  |
| T1 (≤4) | ref | ref | ref |
| T2 (5-6) | 0.72(0.57,0.90) 0.004 | 0.72(0.57,0.90) 0.005 | 0.79(0.61,1.00) 0.053 |
| T3 (≥7) | 0.51(0.39,0.67) <0.0001 | 0.53(0.40,0.70) <0.0001 | 0.66(0.50,0.88) 0.005 |
| *P* for trend | < 0.0001 | < 0.0001 | 0.004 |

This model excludes participates less than 70 years of age.

Model 1: Adjusted for no variables.

Model 2: Adjusted for race, gender, and age.

Model 3: Adjusted for gender, age, race, PIR, education level, marital status, alcohol consumption, smoking status, PA, energy intake.

DI-GM: dietary index for gut microbiota; OR: odds ratio.
